# Safety of localizing epilepsy monitoring intracranial electroencephalograph electrodes using MRI: Radiofrequency-induced heating

**DOI:** 10.1002/jmri.21583

**Published:** 2008-11

**Authors:** David W Carmichael, John S Thornton, Roman Rodionov, Rachel Thornton, Andrew McEvoy, Philip J Allen, Louis Lemieux

**Affiliations:** 1 Department of Clinical and Experimental Epilepsy, Institute of Neurology, University College LondonLondon, United Kingdom; 2 Lysholm Department of Neuroradiology, National Hospital for Neurology and NeurosurgeryLondon, United Kingdom; 3 Victor Horsley Department of Neurosurgery, National Hospital for Neurology and NeurosurgeryLondon, United Kingdom; 4 Department of Clinical Neurophysiology, National Hospital for Neurology and NeurosurgeryLondon, United Kingdom

**Keywords:** safety, intracranial EEG, MRI, electrodes, heating, epilepsy surgery

## Abstract

**Purpose:**

To investigate heating during postimplantation localization of intracranial electroencephalograph (EEG) electrodes by MRI.

**Materials and Methods:**

A phantom patient with a realistic arrangement of electrodes was used to simulate tissue heating during MRI. Measurements were performed using 1.5 Tesla (T) and 3T MRI scanners, using head- and body-transmit RF-coils. Two electrode-lead configurations were assessed: a “standard” condition with external electrode-leads physically separated and a “fault” condition with all lead terminations electrically shorted.

**Results:**

Using a head-transmit–receive coil and a 2.4 W/kg head-average specific absorption rate (SAR) sequence, at 1.5T the maximum temperature change remained within safe limits (<1°C). Under “standard” conditions, we observed greater heating (≤2.0°C) at 3T on one system and similar heating (<1°C) on a second, compared with the 1.5T system. In all cases these temperature maxima occurred at the grid electrode. In the “fault” condition, larger temperature increases were observed at both field strengths, particularly for the depth electrodes. Conversely, with a body-transmit coil at 3T significant heating (+6.4°C) was observed (same sequence, 1.2/0.5 W/kg head/body-average) at the grid electrode under “standard” conditions, substantially exceeding safe limits. These temperature increases neglect perfusion, a major source of heat dissipation in vivo.

**Conclusion:**

MRI for intracranial electrode localization can be performed safely at both 1.5T and 3T provided a head-transmit coil is used, electrode leads are separated, and scanner-reported SARs are limited as determined in advance for specific scanner models, RF coils and implant arrangements. Neglecting these restrictions may result in tissue injury. J. Magn. Reson. Imaging 2008;28:1233–1244. © 2008 Wiley-Liss, Inc.

MRI IS USEFUL for postimplantation localization of intracranial electroencephalograph (EEG) electrodes in epilepsy patients as it allows good visualization of implant positions in relation to neuroanatomy and avoids the ionizing radiation dose associated with computed tomography (CT) imaging.

Implantation procedures can often involve a combination of electrodes of different types, namely subdural grid, strip, and depth electrodes. Strip electrodes consist of a set of disk-shaped electrode contacts imbedded in a silicon sheet that record electrical signals from the cortical surface, while grid electrodes are simply a set of strip electrodes joined together to record EEG signals from a larger area. Depth electrodes are thin rods with cylindrical contacts that penetrate cerebral tissue and can record directly from deep brain structures. Signals from the electrode arrays are recorded by means of several multi-channel flexible connecting leads, referred to herein as “tails.”

As for the case of recording scalp EEG during MRI (1), there are two primary hazards associated with the presence of intracranial EEG electrodes during MRI resulting from induced currents: (i) radiofrequency (RF) -induced heating of tissue surrounding the electrodes, (ii) stimulation of, or destructive current flow in, brain tissue due to switching magnetic gradient fields. Provided the electrode tails are not in contact (to form a low impedance circuit at gradient switching frequencies) then little low frequency current will flow. Furthermore, related electrode implant experiments have confirmed that gradient induced voltages are small (2,3). Motion of the patient relative to the static magnetic field may also induce currents in conducting implants but this effect has been shown not to be hazardous (1). The static magnetic field can also give rise to additional health risks by acting mechanically on (permanently or transiently, e.g., by the passage of current) magnetized conducting materials causing electrode movement or flexion. In this study, we address the most significant safety issue, that is, namely associated with RF heating.

While some intracranial electrodes designed for use in epilepsy monitoring are thought safe for MRI procedures (4–6), and others have been subject to testing (7–12), to our knowledge to date no systematic experimental study addressing the thermal safety of MRI with multiple intracranial EEG electrodes has been published. Two retrospective studies have described clinical observations following MRI in a series of patients with intracranial EEG electrodes, reporting no obvious adverse neurological effects (13,14). These studies provide evidence that the risk of serious injury is low in the specific conditions that were used (i.e., imaging sequence, specific absorption rate [SAR], scanner, RF coil, electrode arrangements, etc). However, they do not demonstrate how to achieve compliance with safety guidelines or determine the specific conditions in which the procedure may be dangerous.

While preliminary in vitro safety testing has also been performed (4,7), these studies used test objects limited in their anatomical realism, the nature of the conductive medium used, which can significantly alter temperature increases (15) and their consideration of the implications of implantation configurations involving multiple, potentially electrically interacting electrode types. Finally, the majority of previous studies were undertaken at 1.5 Tesla (T), while the safety of MRI with these implants at 3T, a field strength used increasingly for clinical examinations, is less well established.

We, therefore, investigated MRI induced heating at 1.5 and 3T in a tissue-simulating test object containing a combination of depth, grid and strip electrodes aiming to replicate a realistic arrangement involving multiple implants. We additionally tested the effects of bilateral depth electrodes unintentionally coming into contact within the tissue, and the effects of electrical contact (short circuit) between the external electrode tails to simulate a worst case “fault” condition. We also considered the choice of head-only or whole-body MRI RF-transmit coil, because MRI systems using the latter are increasingly common.

## MATERIALS AND METHODS

### Test Object and Temperature Measurements

**Figure 1:**
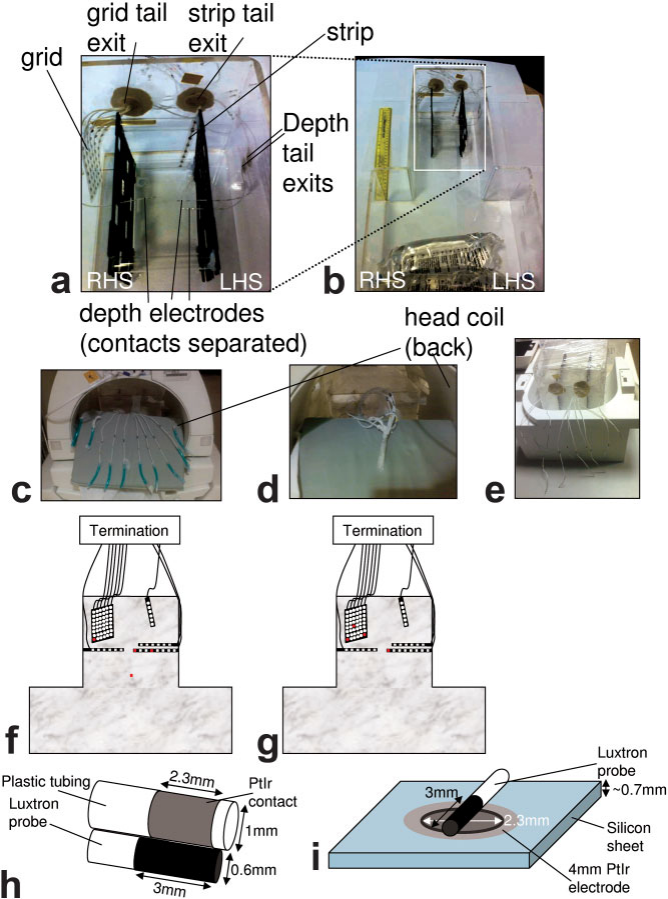
Experimental arrangement without gel. **a**: Head part of the phantom patient shown with the various implants inserted. **b**: Entire phantom with a 30-cm ruler for scale. The phantom patient right hand side (RHS) and left hand side (LHS) are indicated. The black plastic components were used to position and hold the electrodes. **c**,**d**: The two electrode-tail arrangements investigated are also demonstrated with the tails separated in an “open circuit” (c) and bundled together in a “short circuit” termination (d). **e**: The different geometry of the “open circuit” tail arrangement required for the body coil/bottom half of the 12-channel head array coil is demonstrated. **f**,**g**: A schematic figure of the phantom showing the sites of temperature measurement (in red) used for most experiments (f), and for testing temperature changes with grid position where different measurement sites were used (again in red; g). **h**,**i**: The geometry of temperature probes relative to the electrodes is demonstrated for depth (h) and grid contacts (i).

A Perspex phantom, similar to that described in Finelli et al (3,16), was formed with a shape and dimensions approximating those of an adult human torso (Fig. 1), and filled to a depth of approximately 10 cm with a semiliquid gel comprising distilled water, polyacrylic acid partial sodium salt (Aldrich Chemical; 8 g/L) and sodium chloride (0.70 g/L) with electrical and thermal characteristics similar to those of human tissue (15,16).

The specific electrodes tested were those commonly used at our own and many other institutions for intracranial EEG monitoring in patients with epilepsy. The depth electrodes (Ad-Tech, Racine, WI) consist of Platinum contacts of 2.3-mm length with a radius of 1 mm, with nickel–chromium wires in polyurethane tubing leading to nickel–chromium tail contacts. There are a range of electrodes available which vary in terms of numbers of contacts and their separation. The electrodes used here were 1xSD-8PX (8 contact electrode with 10-mm spacing, total length 380 mm), 2xSD-6PX (6 contact electrode with 10-mm spacing, total length 370 mm). Grid and strip electrodes (Ad-Tech, Racine, WI) have 4-mm diameter PtIr disks (2.3 mm exposed) within a silicon sheet with stainless steel (316) wires and nickel–chromium tail contacts contained within polyurethane tubing. The strip used here was a T-WS-6PX (6-contact electrode with 10-mm spacing, total length 445 mm), the grid was a T-WS-48PX (6 × 8 contacts with 10-mm spacing, and 6 tails, total length 455 mm).

For all the experiments, 3 depth electrodes, 1 subdural grid, and 1 strip electrode were positioned within the head phantom (Fig. 1). The depth electrodes were inserted along the left–right axis and perpendicularly to the sagittal plane, two on the left hand side (LHS) (1 × 8 contacts, 1 × 6 contacts) and one on the right hand side (RHS) (1 × 6 contacts). This simulated implants targeting the left hippocampus and amygdala with contra-lateral control. The electrodes' leads were run along the phantom wall (within the gel) for 40 mm before exiting the phantom to simulate surgical implantation with electrodes tunneled under the skin away from the cranial window to avoid infection. The lengths inside/outside the phantom for the grid electrode were 155/300 mm, for the strip electrode 95/350 mm, for the RHS depth electrode 120/250 mm, for the LHS 6 contact electrode 105/265 mm, for the LHS 8 contact electrode 115/265 mm. The subdural grid and strip electrode were placed in a configuration that simulated implants recording from the cortical surface. This configuration of the electrodes within the gel is hereafter termed “normal” and was tested with each of the three scanners; specific experiments with changes to this configuration are described in detail below.

In all cases, measurements were performed with 2 different electrode tail arrangements: (a) physically separated such that the terminations were an “open circuit”, as per the manufacturer's recommendation (Fig. 1c), to simulate a “standard condition”, and (b) bundled together and secured such that the terminations formed a short circuit to simulate a “fault condition” (Fig. 1d). Except where specifically detailed below, the 8 contact depth electrode was used as the central landmark to be positioned at the scanner isocenter when prescribing the image geometry.

Continuous temperature measurements were made simultaneously from 4 positions using an MRI-compatible fluoroptic thermometer (Model 3100, Luxtron Corporation, Santa Clara, CA; accuracy ±0.1°C; SMM probes) at a rate of 0.5 Hz. A period from 1 min before to 4 min after the 6-min duration scan was used to determine maximum temperature changes. The tips of electrodes are generally considered the locations most likely to demonstrate the largest temperature change (17,18). To further confirm this we performed several pilot experiments placing the sensors at the point of entry of the electrodes into the gel, the strip electrode, the grid and depth electrodes. We also used preliminary (unpublished) modeling results to inform our choice of temperature recording sites. The temperature-sensor fiber tips were placed such that they lay in a transverse position relative to the electrode contacts (see Fig. 1h,i). Temperature measurement sites were modified for some experiments and so are given in each section below.

All MRI sequence SAR values given below are those reported by the software of the particular system at scan time with an entered patient weight of 50 Kg. The limitations and implications of this approach are addressed in the discussion section.

### 1.5T Imaging

Measurements were performed in a 1.5T GE Signa MRI system (software level lx 9.1; GE Healthcare, Milwaukee, WI) with the standard transmit–receive birdcage head coil. A 6-min fast spin-echo (FSE) acquisition with a SAR value of 2.4 W/Kg (head average) was used to elicit the highest temperature changes likely in a structural imaging study. Sequence details were as follows: repetition time (TR) 4660 ms; echo time (TE) 104.4 ms; bandwidth (BW) 31.2 kHz; field of view (FOV) 24 × 18 cm; matrix 256 × 224; echo-train length (ETL) 24; 25 slices; slice thickness (ST) 5 mm; slice separation (SS) 1 mm; 8 averages. The temperature probes were sited at the following locations: the most distal (contact #1) and middle (contact #4) contacts of the 8-contact depth electrode on the LHS, the corner of the grid (contact #48) and at a reference position within the neck region of the phantom away from all electrodes (see schematic Fig. 1f).

To assess the reproducibility of the temperature recordings, measurements were repeated on a further four separate occasions with all electrodes in the “normal” configuration, in each case the temperature sensors and electrodes being repositioned as accurately as possible in the locations described above.

In addition to the “normal” electrode arrangement, with spatial separation between all depth and strip/grid electrodes within the gel, a further configuration was used to investigate the effect of electrodes coming into direct contact within the tissue. The depth electrodes were repositioned such that the RHS 6-contact depth electrode and the LHS 8-contact depth electrode most distal contacts were in direct contact while positioned parallel and adjacent to each other. Furthermore, the temperature probes were repositioned such that they were adjacent to these contacts (one sensor for each of the touching depth electrode contacts) on the opposite side to the part of the electrode-contacts which were touching. The other two sensors remained at the grid corner (contact #48) and at a reference position within the neck region.

### 3T Imaging: General Electric 3T Excite

Measurements were performed in a GE 3T Excite system (software level 12_M4) using the standard transmit–receive birdcage head coil provided. A 6-min FSE acquisition with a SAR of 2.5 W/Kg (head-coil average), similar to that used at 1.5T, was used. Sequence details were as follows: TR 6000; TE 102; BW 31.5 kHz; FOV 22 × 22; matrix 512 × 256; 17 slices; ST 5 mm; SS 1.5 mm; averages 2. Again temperatures were recorded continuously during acquisition; in this case, only the “normal” intracranial electrode arrangement was investigated. Temperature probes were sited at the following positions: the most distal (contact #1) and middle (contact #4) contact of the 8-contact depth electrode on the LHS, the corner of the grid (contact #48) and at a reference position within the neck region of the phantom away from all electrodes (see schematic Fig. 1f).

### 3T Imaging: Siemens 3T TIM Trio

Measurements were performed in a Siemens 3T TIM Trio MRI system (software level VB13 SP2; Siemens AG, Erlangen, Germany) using, first, a transmit–receive birdcage head coil (USA instruments, Aurora, Ohio), and second, the manufacturer-supplied body-transmit coil together with the posterior half of a 12-element head-receive coil for signal reception. A high-SAR, 6-min FSE imaging sequence similar to that used with the GE systems was used. Sequence details were as follows: TR 6000 ms; TE 106 ms; BW 81.9 kHz; FOV 20 × 20 cm; 20% PE oversampling; matrix 512 × 410; 13–18 slices; ST 3 mm; SS 0.3 mm; NEX 2, ETL (Turbo factor) 17. This gave reported SARs of 2.4 W/Kg head-average for the head coil and 1.2 W/Kg exposed volume-average for the body coil with an identically prescribed protocol. One additional measurement was performed with the body coil in which SAR was maximized by using the maximum permitted number of slices within the TR together with an additional magnetization-transfer pulse achieving an exposed body-average SAR of 2.0 W/Kg with the body coil.

In contradistinction to the GE MRI systems, which apparently estimate SARs solely based on the patient weight entered at the console, the Siemens system SAR estimation incorporates a measurement of patient-specific coil loading. Consequently it was found that the SAR values reported by the Siemens system software were quite dependant on the exact phantom position and electrode configuration. For consistency, for each head-coil measurement the number of slices was varied (between 13 and 18) to achieve a reported head-average SAR of 2.4 ± 0.1 W/Kg.

Again temperatures were recorded continuously during acquisition, in this case only the “normal” intracranial electrode arrangement being investigated and were sited at the following locations: the most distal (contact #1) and middle (contact #4) contacts of the 8-contact depth electrode on the LHS, the corner of the grid (contact #48) and at a reference position within the neck region of the phantom away from all electrodes (see schematic Fig. 1f).

### Effects of Scanner Landmark and Grid Implant Positions (Siemens 3T TIM Trio)

First, the effect of landmark position (to assess the impact of different patient positions relative to the magnet isocenter and body RF coil) was examined by running the FSE sequence (exposed body-average SAR 1.2 W/Kg) with the landmark centered on the 8-contact depth electrode and subsequently with offsets of ±100 mm along the scanner bore. These measurements were performed with the body-transmit coil and the electrode-tails bundled together in electrical contact. Temperature measurement locations were the most distal (contact #1) and middle (contact #4) contacts of the 8-contact depth electrode on the LHS, the corner of the grid (contact #48) and at a reference position within the neck region of the phantom away from all electrodes (see schematic Fig. 1f).

**Figure 2:**
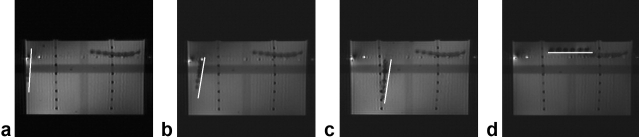
Visualization of the different grid positions tested. The white bars are adjacent to the grid position as seen in an axial scout image. **a**–**d**: Parts correspond to the grid electrode positions a–d as reported in Table 4.

Second, the effect of adjusting the position of the grid electrode-array within the phantom was investigated using four different arrangements with different positions and orientations (see Fig. 2): (a) with the grid array adjacent to the wall of the phantom, (b) with a position typical of those used in the previously described measurements, that is, separated from the phantom wall by approximately 1 cm, (c) the opposite side of the spacer bars (see Fig. 1), and (d) with the grid rotated through 90° to lie in the coronal plane. In experiment (d) the temperature was measured from three of the grid contacts (the two corners, contacts #1 and #48, and one more central, contact #20, see schematic Fig. 1g). These measurements were performed with the head-transmit coil and the electrode-tails bundled together in electrical contact.

**Table 4:** Maximum Temperature Changes for Different Grid Positions Within the Phantom Using the Head Coil / 3T Siemens System

Grid position	ΔT °C			
	Depth (contact #1)	Grid (contact #1)	Grid (contact #20)	Grid (contact #48)
a	0.4	0.2	0.2	0.4
b	0.4	0.3	0.3	0.7
c	0.3	0.4	0.2	0.5
d	0.7	0.7	0.5	2.0

## RESULTS

### 1.5T Imaging

In all cases with more than one temperature sensor on an implant, the values from the site of greatest temperature change are reported.

#### Temperature Change Reproducibility

The results of conducting the same experiment 5 times on separate days, requiring the phantom, temperature probes and electrodes to be repositioned relative to each other and the scanner are shown in Table 1. Experiments 4–5 were performed sometime after experiments 1–3 requiring a new gel solution of the same composition to be used. The mean and range of the measurements from the grid and depth electrodes with the two configurations: (a) tails physically separated, open circuit, (b) tails bundled together, short circuit, demonstrated that grid electrode temperature increases (ΔTs) were consistent between measurements in both configurations but showed a greater range in the shorted condition (+1.2 to +1.7°C). For the depth electrode, there was consistently no measurable ΔT (always < 0.1°C) provided the tails were separated; however, larger increases and a greater range of ΔTs were obtained when the tails were short circuited (+0.9 to +3.0 °C).

**Table 1:** Maximum Temperature Changes for Repeated Measurements Using the 1.5T GE With Head Transmit RF Coil

Experiment #	Tail termination	ΔT °C	
		Depth	Grid
1	Open circuit	<0.1	0.7
	Short circuit	1.4	1.4
2	Open circuit	<0.1	0.7
	Short circuit	1.8	1.7
3	Open circuit	<0.1	0.7
	Short circuit	2.5	1.5
4	Open circuit	<0.1	0.7
	Short circuit	3.0	1.3
5	Open circuit	<0.1	0.8
	Short circuit	0.9	1.2
Mean	Open circuit	<0.1	0.7
	Short circuit	1.9	1.5
Range	Short circuit	<0.1	0.7-0.8
	Open circuit	0.9-3.0	1.2-1.7

#### Head-Transmit Coil

**Figure 3:**
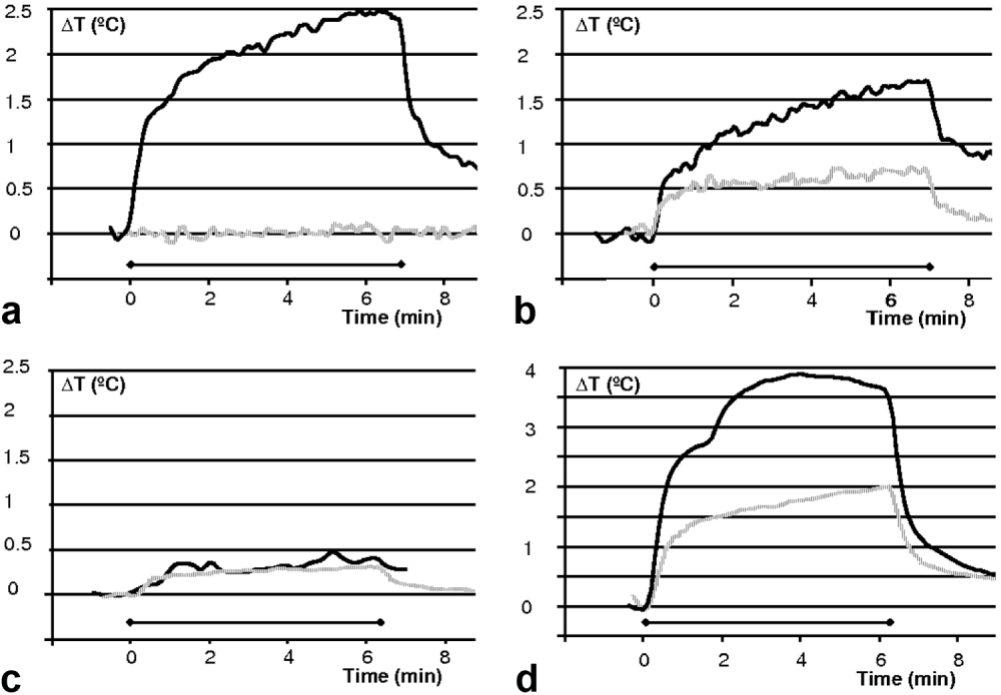
Maximum temperature changes at 1.5 and 3T. Dark solid line: electrode tails shorted together; gray dotted line: electrode tails electrically isolated and separated. Horizontal gridlines are always 0.5°C and the horizontal bar indicates the scan period. **a**: 1.5T GE system, head-transmit coil, depth electrode. **b**: 1.5T GE system, head coil, grid electrode. **c**: 3T GE system, head coil, depth electrode. **d**: 3T GE system, head coil, grid electrode.

With the electrode tails separated, the maximum ΔT at all measurement points was always < 1°C (Table 2; Fig. 3a,b). The depth electrode did not show any heating whereas the grid electrode demonstrated a modest temperature increase (+0.7 to +0.8°C). Maximum ΔT was always increased by shorting the electrode tails at both the grid and depth electrodes. The difference in maximum ΔT between the two electrode tail configurations (i.e., open versus short circuit) was smaller for the grid electrode compared with the depth electrode.

**Table 2:** Maximum Temprature Changes for Different Scanners,RF Coils and Electrode Arragement.

Scanner[coil]	SAR(W/Kg) head/body	Tail termination	ΔT°C	
			Depth	Grid
GE 1.5T [Head coil]	2.4/0.2	open circuit	<0.1	0.7
		short circuit	2.5	1.7
repeated with depth electrode tips touching		open circuit	<0.1	0.7
		short circuit	1.9	1.7
GE 3T[Head coil]	2.5/0.2	open circuit	0.3	2.0
		short circuit	0.5	3.9
Siemens 3T [Head coil]	2.4/0.2	open circuit	0.2	0.9
		short circuit	1.6	1.7
Siemens 3T [Body coil]	1.2/0.5	open circuit	0.7	6.4
		short circuit	0.5	0.7
	2.0/0.8	short circuit	0.8	1.2

#### Depth Electrodes Touching Within the Gel

Very similar results to those above were obtained for the second configuration of the depth electrodes where the electrode tips were in direct contact within the gel (compare 1.5T results in Table 2). In this experiment temperature was recorded from the opposite sides of the touching contacts from the left and right hand side depth electrodes. With the electrode tails in the open circuit configuration neither depth electrode showed a significant ΔT (<0.1°C). Both electrodes showed a significant increase when the tails were shorted with the LHS electrode heating to +1.9°C and the RHS electrode to +1.1°C.

### 3T Imaging: GE 3T Signa Excite system

#### Head-Transmit Coil

The grid electrode was the site of greatest temperature increase (+2.0°C) in the open circuit tail configuration (Table 2; Fig. 3d) with a smaller ΔT seen at the depth electrode (+0.3°C, Fig. 3c). As at 1.5T, ΔT was always increased by shorting the electrode tails, with the greatest ΔT (+3.9°C) occurring at the grid electrode. The largest recorded temperature increase (+6.9°C) was obtained during a pilot experiment on the GE system with the tails shorted and while acquiring a higher SAR (3.8 W/Kg head-average) scan which was interrupted automatically due to the scanner's time-averaged SAR limit (3 W/Kg for 6 min) being exceeded.

### 3T Imaging: Siemens 3T TIM Trio System

#### Head-Transmit Coil

**Figure 4:**
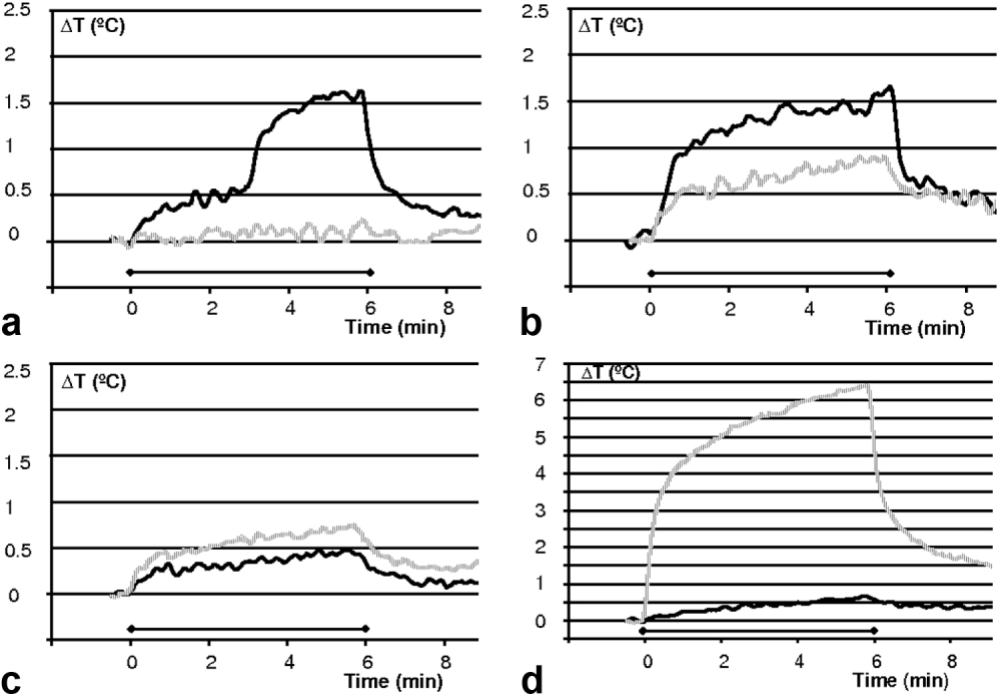
Maximum temperature changes using different RF-transmit coil arrangements at 3T. Dark solid line: electrode tails shorted together; gray dotted line: electrode tails electrically isolated and separated. Horizontal gridlines are always 0.5°C and the horizontal bar indicates the scan period. All data are from a 3T Siemens TIM Trio system. **a**: Head coil, depth electrode. **b**: Head coil, grid electrode. **c**: Body coil, depth electrode. **d**: Body coil, grid electrode.

With the electrode tails shorted the maximum ΔT was +1.7°C for the grid and +1.6°C for the depth electrode (Fig. 4a,b). Again, with the tails-separated open-circuit termination, the maximum ΔT was reduced (+0.9°C) for the grid, and by a greater margin for the depth electrode (+0.2°C).

#### Body-Transmit Coil

The greatest ΔT (+6.4°C) occurred at the grid electrode when the electrode tails were separated (i.e., opposite to the effect seen with the head-transmit coil; Fig. 4d). At the depth electrode, markedly lower ΔT were observed (+0.7°C, Fig. 4c); however, this was by far the greatest temperature increase recorded at the depth electrode in the open circuit configuration. With the electrode tails shorted, the maximum ΔT at the grid and the depth electrodes was reduced to +0.7°C and +0.5°C at each implant, respectively. These temperature changes were produced using the same scan protocol as for the head coil measurements. A further body-coil transmit scan was run with increased SAR (see Table 2, bottom row) with the tails shorted; this induced greater heating proportional to the increase in SAR but ΔT was still small in comparison to that obtained with the open circuit tail termination and the lower SAR protocol.

#### Effects of Scanner Landmark and Grid Implant Positions

Three different landmark positions were tested (see Table 3) while running an identical protocol. There were differences in the peak temperature changes, with higher values seen at all the measured electrode positions for the table position 100 mm further into the scanner bore (maximally +0.7°C for the depth, +0.9°C for the grid) compared with those obtained with the table centered (maximally +0.4°C for the depth, +0.6°C for the grid). However with the table landmark −100 mm (100 mm less far into the scanner), the results were very similar to those obtained with the central landmark (maximally +0.4°C for the depth, +0.7°C for the grid), despite the scanner reported SAR increasing from 1.2/0.5 W/Kg to 1.4/0.9 W/Kg. It is of note that the scanner-calculated SAR changed substantially between landmark positions, despite an otherwise identical sequence, due to changes in the scanner coil-loading and the scanner estimates of exposed mass with position relative to the RF coil. All ΔT were modest for this arrangement (body-transmit coil; electrode tails shorted externally).

**Table 3:** Maximum Temperature Changes for Different Landmark Positions Using the Body Coil / 3T Siemens System

Table position (mm)	ΔT °C				SAR (W/Kg)
	Depth (1)	Depth (2)	Ambient	Grid	head/body
100	0.6	0.7	0.2	0.9	1.2/ 0.5
−100	0.3	0.4	0.1	0.7	1.4/ 0.9
0	0.3	0.4	0.2	0.6	1.2/ 0.5

The peak temperature change varied significantly as a function of grid electrode position and orientation. The greatest temperature change (+2.0°C) occurred with the grid arranged horizontally near the gel surface (see Fig. 2; Table 4). The corner of the grid electrode was the site of greatest temperature change. Interestingly the temperature change measured at the depth electrode also changed with grid position highlighting that there was an interaction that alters with implant position. We note that these experiments were performed with the electrodes tails shorted where variability in the results was found due to the exact manner in which the electrode tails were shorted. The maximum recorded temperature change in any of the positions was not greater than that measured previously.

## DISCUSSION

It is well established that exposure of electrodes and leads to the RF electromagnetic field in MRI can give rise to a concentration of the electric field in surrounding conductive tissue (19). This results in a locally elevated specific absorption rate (SAR) which may cause locally increased RF-induced heating. Therefore, similar to the cases of scalp EEG (1) and deep-brain stimulation (DBS) electrodes (2,3,16,20–22), the limiting of RF-induced heating to acceptable levels is of primary concern. Our study, therefore, addressed MRI safety only with respect to the RF-induced heating associated with these particular electrode types.

### RF-Related Guidelines, Limits, and Device Labeling

Current international guidelines (23) recommend that MRI-induced heating should not cause temperature in the head to exceed 38°C, suggesting an allowable increase of <1°C. The guidelines also specify MRI SAR limits intended to restrict tissue temperature increases to within these levels, which only apply in implant-free patients. SAR limits are specified in addition to temperature increase limits due to the practical difficulties associated with accurate determination or prediction of local tissue temperature increases in vivo. The head-average SAR limits are 3.2 W/Kg for a 6-min exposure period (23) or 3 W/Kg for a 5-min exposure period (FDA) (6). The local SAR should not exceed 10 W/Kg averaged over 10 g of tissue (IEC) (23) or 8 W/Kg over 1 g of tissue (FDA) (6). The manufacturer of the electrodes used in this work (Ad-Tech Medical) does not have, or claim FDA approved MR-Conditional status for these products, or European certification for safe use with MRI. However (otherwise unpublished) test results for these products are reported in a statement provided by the company (9) suggesting that MRI may be safe under certain specific conditions, including the recommendation that the electrode tails are separated to ensure electrical isolation.

### SAR as a Dosimeter When Implants Are Present

Commercial MRI systems report local maximum, head-, and or body-average SAR estimates for all acquisition sequences based either on theoretical calculation, or measurements of the total RF power absorbed by the subject. The purpose of these estimates is to ensure that the RF power deposition during clinical MRI examinations of implant-free patients lies within the safe limits. In general they represent an upper bound and have been found in some cases to overestimate the true volume-average power deposition by an unknown factor (24), whereas local values may be underestimated (25). The exact methods for estimating and reporting SAR values may vary between different models and manufacturers of MRI equipment. Therefore, absolute relationships between scanner-reported SAR and tissue temperature elevation are not directly transferable between MRI systems or between the same system running different software versions (26,27). Additionally, operator-entered parameters such as patient weight and system-determined parameters such as coil input power can affect the reported SAR for a specific scanner model and software (1,26). Hence, when determining SAR-based safety limits the uncertainty in the scanner reported volume average SAR should be considered.

Despite the above-mentioned limitations of scanner-reported SAR as a dosimeter, it is nonetheless currently the only available appropriate index (28) to determine the types of MRI protocol that carry acceptable risk and to guide local safety assessments. We have, therefore, followed this convention but with the caveat that the results obtained in this study are only directly relevant to the scanners and software versions tested: translation to other scanners or software versions will require cross-platform validation.

The effect of elongated conducting implants within the RF field of the MRI system is to concentrate the electric RF field at certain locations proximal to the implant causing the local tissue power deposition to increase. The assumptions used to model local SAR by the scanner software are not valid when an implant is present, for example, scanner-reported head-average and local SARs, which would be safe for nonimplant patients, may nevertheless cause thermal injury in the presence of intracranial electrodes, with the local SAR estimate from the scanner, therefore, inaccurate. Hence, the volume average SAR must be limited to prevent local heating exceeding guideline levels.

Many in vitro implant studies have shown that temperature increases linearly with scanner-reported volume average SAR (2,26,29) and crucially the intra-scanner reported SAR values produce temperature rises according to a consistent linear relationship for different pulse sequences and parameters with high reliability (26) given an equivalent scan duration. Therefore, it is rational to specify a maximum safe scanner-reported SAR level for imaging these patients, understanding that the recommended maximum value applies only to the specific equipment and range of circumstances tested.

In light of the above, we used the established methodology of measuring local in vitro temperature changes using MR sequences with scanner-reported SARs approaching the IEC limits (3.2 W/Kg head SAR, averaged over 6 min) (23) and exceeding the long duration uncontrolled limit (2 W/Kg, head average, >30 min) (30) normally adhered to locally for clinical neuroimaging. Our measurements were performed on three different MRI instruments, and with different RF-transmit–receive coil arrangements to derive general safety principles and, as appropriate, some MRI equipment-specific SAR limits using the established intra-scanner relationship between SAR and local temperature changes.

### 1.5T GE System With Head-transmit Coil

#### Measurement Reproducibility

Maximum ΔT were highly reproducible with the electrode tails separated (see Table 1). The last two measurements in this table were obtained over a year from the first three requiring complete repositioning of the electrodes, temperature probe locations, and new gel. Thus confirming that with the electrodes separated as in Figure 1c, the temperature changes were consistent and predictable to within the accuracy of the temperature measurement probes and background temperature fluctuations.

Reproducibility was not as high in the “fault condition,” suggesting that the exact way that the tails are bundled together introduces significant variance by altering both the degree of contact made between electrodes and the area of conducting loops thus created. Of interest, in two measurements with the electrode tails shorted (e.g., Fig. 4a) there was a period during scanning when the rate of temperature increase changed, suggesting a change in the electrical properties of the configuration. We postulate that vibration-induced changes in the inter-electrode tail contact configuration in the short-circuit condition were responsible. This was confirmed by re-running the experiment and altering how the electrode tails were in contact by squeezing them together during a scan, at which time a step change in the rate of temperature increase was recorded.

These observations show that with a careful and consistent arrangement of the electrode tails such that they are not in electrical contact with each other, and are positioned away from the end rings of the RF coil (Fig. 1c), the temperature changes are highly reproducible.

#### Head Coil

With the electrodes and leads arranged consistently with the manufacturer's recommendation, that is, with the tails physically separated we did not observe heating above the guideline level confirming that this arrangement is safe under these specific conditions (GE 1.5T Signa 9.1lx MRI system with head-transmit–receive coil and the electrode tails separated). However, other configurations led to temperature increases which exceed safety limits: Electrically short-circuiting the electrode tail terminations produced greater temperature increases, up to +3.0°C for the depth electrode in the fourth reproducibility measurement. Conversely, shorting opposing contra lateral depth electrode contacts within the gel did not significant alter ΔT at 1.5T, which is attributable to the electrodes being effectively connected through the tissue simulating gel even when not in direct physical contact.

The subdural grid electrodes demonstrated ΔT of up to +0.7°C even in the open-circuit tail configuration suggesting that they are more strongly coupled to the RF field during “standard operation,” and, therefore, with a head-transmit RF coil, they present the greatest risk of excessive heating.

Whereas the overall temperature increases observed at 1.5T may be considered modest, it remains prudent to minimize the risk of excessive heating beyond safety guidelines due to unforeseen experimental circumstances by restricting the scanner-reported SAR of the MRI sequences used, by using a head-transmit coil only, and by rigorously maintaining the separation of the electrode tails as in Figure 1c.

### 3T Imaging: GE 3T Signa Excite

#### Head Coil

Although overall heating was greater for the 3T GE scanner than for the 1.5T GE system, the pattern was very similar, with the greatest ΔT occurring with the tails shorted. In a pilot study with this “fault” condition, where a higher SAR sequence (3.8 W/Kg coil average) was used, heating to +6.9°C was recorded. It is, therefore, clear that without proper precautions potentially injurious ΔT may occur. In the open-circuit tail configuration, the grid electrode was again the site of greatest ΔT (+2.0°C). With this scanner it was possible to elicit ΔT above the guidelines with both electrode tail configurations: to ensure patient safety in this case scanner-reported SAR should be limited to significantly less than 2.5 W/kg head-average.

### 3T Imaging: Siemens 3T TIM Trio

#### Head Coil

Temperature increases were less than for the 3T GE system but again the pattern was similar, with the greatest ΔT, sufficient to exceed safety guidelines, occurring with the tails shorted. The grid electrode was again the site of greatest temperature increase (+0.9°C) with the open circuit tail configuration.

Whereas the overall temperature increases observed with this scanner may also be considered modest in the specific case of using a head-transmit coil, the risk of tissue heating beyond safety guidelines due to unanticipated events may again be reduced by minimizing SAR and maintaining the electrical isolation of the electrode tails.

#### Body Coil

For the Siemens 3T body-transmit coil, the greatest ΔT (+6.4°C) occurred at the grid electrode when the electrode tails were separated, that is, under standard operating conditions. This level of heating far exceeds guidelines and approaches the level at which permanent tissue-damage could occur rapidly. Furthermore, with this coil it would be theoretically possible to prescribe scans with significantly greater SAR causing even higher ΔT. Markedly lower temperature increases (+0.7°C) were observed at the depth electrode, but again this was obtained with the electrode tails separated. Shorting the electrodes tails resulted in lower ΔT at all measurement points.

These results suggest that when the entire length of the electrodes (including the tails) lies within the RF field, as occurs when using the body-transmit coil, interactions resulting in greater heating may occur. This is possibly due to a larger effective cross-sectional area of the circuit exposed to the body coil's RF field in the open circuit compared with that with the shorted-tail configuration. Also, due to the geometry of the head receive-only coil (Fig. 1e) used in this case, the open-circuit tail arrangement was slightly altered in comparison with the head-transmit coil experiments (Fig. 1e), possibly causing stronger coupling to the body coil's electric field. While the smaller ΔT seen with the electrodes electrically shorted might seem to suggest that it is possible to safely image these implanted patients with the body-transmit coil with such a configuration, we note the following: the variability of the ΔT obtained with the tails shorted was high and in some measurements the exact degree of contact between electrode tails appeared critical; variability in landmark position can lead to different heating with this arrangement (see below); and due to the creation of a low-impedance conducting loop, the opportunity for gradient induced tissue stimulation or necrosis may arise. Hence, in practice we do not recommend that the tails are shorted together. Further experiments would be required to assess whether safety could be improved by reducing the exposed circuit area (i.e., by twisting the electrode tails together while maintaining electrical isolation of the terminations). In conclusion, the use of body RF-transmit coils presents a significant risk of injury and is not advised.

#### Effect of Scanner Landmark Position

With the body-transmit coil, the dependence of ΔT upon landmark position (Table 3) suggested that, due to spatial variations in the RF-field, the exact temperature increase may change. Additionally, the patient position relative to the body RF coil produced significant differences in scanner estimated SAR (with an increase in head/body SAR from 1.2/0.5 W/Kg for landmarks at 0 and +100 mm to 1.4/0.9 W/Kg at the −100 mm position). The temperature increases did not change proportionally to these SAR differences. It is clear from the limited measurements obtained at different landmark positions that the use of a body-transmit coil introduces considerable extra uncertainty in the scanner-reported SAR and resulting ΔT for a particular pulse sequence. This effect does not arise when head-transmit RF coils are used because in this case the patient position relative to the coil is fixed. For this reason the use of head-transmit coils is recommended.

#### Effect of Grid Implant Position

Changing the position of the grid within the phantom had a significant effect on ΔT: a fivefold increase in heating was recorded with the grid in a superficial position in the coronal plane compared with the other, sagittal orientations tested. This suggested that significant variations in heating can occur as a function of implant position and, or, orientation. The overall temperature changes in both cases remained modest (maximum +2.0°C) in these experiments with shorted electrode tails. However, our observations support the argument that either detailed, implant-arrangement specific tests are required, or that a suitable safety margin to account for electrode-orientation dependent variations should be factored in when local safe upper-SAR limits are defined.

### Experimental Considerations

Our measurements broadly follow the principles of ASTM F 2182-02a (31), a standard for testing MRI-induced temperature increases near passive implants, which has also been used for similar testing of elongated implants leading to FDA certification (28). However, the distribution of the electric and magnetic fields within our phantom may not accurately represent the exact field distribution found within a human body comprised of numerous tissue compartments with differing electrical properties. This makes representations of absolute “worst case” or “typical” implant positioning difficult (24,32); furthermore, the MRI-implant interactions for a particular electrode configuration may vary with scanner, coil and field strength (24). Despite these potential limitations, we followed the best available published methodology for testing elongated implants by using a clinically relevant arrangement, and then sought to test the variability in these measurements (28) due to altered electrode tail arrangements, altered grid–electrode position within the phantom and different scanner and coil combinations.

We believe our experimental parameters were such as to provide realistic upper limits for ΔT: The gel phantom is a conservative model for tissue heating; it is expected that ΔTs would be smaller in vivo, because brain temperature is cooled by perfusion (33,34); a large number of electrodes were tested simultaneously to increase the possibility of resonant loop and/or antenna formation; the electrode tails were shorted to test a “fault condition” where conductive loops are formed; a pulse sequence was chosen to provide a head-average SAR close to the statutory limits.

The EEG electrodes were arranged in a configuration typical of surgical procedures performed at our institution. The potential for underestimation of ΔT due to experimental error was investigated by performing several experiments: a repeated measurement without moving the implant position; a repeated measurement while moving the electrode grid; changing landmark position (testing altered position within the body coil). We have shown that moving the electrode grid within the test object, and variations in landmark position, did indeed cause changes in ΔT. Experimenters should be aware that such changes may easily arise as a result of variations in implant positioning between individual patients, or deviations from a standard supine patient position relative to the scanner bore.

Potential inaccuracies exist in studies of this type relating to the precise positioning of the temperature-sensing optical-fiber tips (35). We positioned temperature probes in a transverse configuration relative to the electrode contacts (Fig. 1), a placement method which gave the highest peak temperature change for pacemaker electrodes (35). Repeated measurements then confirmed that provided the electrode tails were separated the observed ΔT were highly reproducible. The main source of variability was found to be due largely to small changes in the degree of electrical contact between the shorted tail terminations.

For the depth electrodes, maximum ΔTs were similar at the tip and fourth electrode contact in most experiments with greater variance between the positions when the electrodes were shorted. This finding suggests that significant ΔTs are not restricted to the tip electrode contact as might be suggested by simple models (17,18). Pilot studies demonstrated that little heating was observed in the gel adjacent to the wire exit points and that the strip electrode behaved very similarly to the depth electrodes. Whereas we believe that the ΔTs we report are representative, measurement locations were chosen on the basis of these pilot experiments and preliminary modeling results (unpublished observation), we cannot exclude the possibility that greater heating may have occurred at electrode positions we were unable to monitor.

The heating in excess of safety guidelines reported here was produced using a high SAR FSE sequence prescribed specifically to obtain large ΔT. Such sequences, used here for experimental purposes, should not be used to image patients with these implants. A 6-min imaging sequence was chosen because it provides sufficient time to acquire most standard structural imaging data sets and corresponds to the duration of the IEC SAR limit (23). Although the largest rate of temperature change occurs within the first minute of scanning, longer duration sequences may cause greater temperature increases and hence require further evaluation. Furthermore, a “fault condition” with the electrode tails being shorted was used to try to obtain an upper limit for the range of likely ΔT. Despite our observation of reduced heating in one body coil experiment with such an arrangement, we suggest that, in accordance with the manufacturer's recommendations, this configuration should be avoided in vivo; the formation of electrically conductive loops is known to pose a safety risk (1,19).

Our results are consistent with those obtained previously: MRI with a single depth electrode resulted in little measurable heating at 3T (7,9,11), as we found for our most similar arrangement, that is, ΔT at the depth electrode with the open-circuit tail termination and the head-transmit coil. We note that with more than one electrode present, conditions can exist which dramatically alter heating (e.g., shorted tails). Previous testing of grid electrodes also resulted in little heating (4), in this case with the grid electrode tails looped and entirely implanted within gel. We note that, although the latter arrangement may seem to maximize loop area exposed to the RF field, it results in a change in the resonant electrical length, and, therefore, may significantly alter frequency-dependent RF interaction relative to the in vivo situation.

### Defining Safe Protocols

For the practical purpose of defining an imaging protocol on a specific scanner we locally determine an SAR limit based on the expected temperature changes for specific implantation configuration (see the SAR as a Dosimeter When Implants Are Present section). We include a fivefold safety margin to account for intra-scanner dependent variability in SAR estimation from patient dependent variations in implant position, patient weight, patient position and tissue distribution. This suggests that the expected temperature increase should not exceed +0.2°C at any location in our experiments. In the “fault condition” the maximum heating should also be limited to <1°C and the most conservative SAR value used. Taking the example of the experiment performed in this work using the 1.5T GE system, with the head-transmit coil where a maximum temperature increase of +0.7°C was recorded with a 2.4 W/Kg sequence in the “standard” condition (i.e., the electrodes tails separated and arranged as in Fig. 1c), we would recommend limiting the SAR to 0.7 W/Kg (head average, 6 min average).

Generalization of these results to other MRI equipment will require further system-specific experiments. We reiterate that SAR limits for safe MRI in the presence of intracranial electrodes cannot be easily generalized across MRI scanners without careful cross-scanner calibration (26,27). In particular, we note that even if calorimetric measurements were performed to cross-calibrate scanner-reported average SAR between systems, then hardware-dependent variations in RF-field interactions might still cause significant differences in the resulting temperature increases. A local safety assessment and strict adherence to a fixed experimental protocol is, therefore, essential if MRI is to be performed safely in subjects with implants such as those required for intracranial EEG recording.

In particular we recommend that only head-transmit RF coils are used, that electrode tails are carefully isolated, arranged in a consistent position that has been tested and that local scanner-, software-, and RF coil-specific head-average SAR limits are determined and strictly followed. A conservative margin of safety to account for any experimental uncertainty such as the accuracy of temperature measurement and inter-patient variations in EEG-electrode placement and scanner reported SAR is prudent.

In conclusion, in the absence of proper safety precautions, MRI for localizing intra-cranial EEG electrodes poses a significant risk RF-induced thermal injury. However, such procedures can be performed safely at both 1.5T and 3T providing a head-transmit coil is used, electrode tails are separated and appropriate experimentally determined SAR limits are observed.

We emphasize that, while we believe our measurements are representative of our local surgical and imaging practices, any changes in the electrode implantation configuration (e.g., different lead geometry or implant type), or changes in the subject's exact position within the scanner may result in different heating patterns. Therefore, arrangements which substantially differ from these may require further specific safety investigation, as may modifications of the imaging protocol, such as a increasing the scan duration or number of slices. In any case, when setting local SAR limits, it is prudent to allow a conservative safety margin to avoid inter-patient variations in EEG-electrode placement, scan prescriptions or RF coil loading causing excessive tissue heating.
